# Thermal, Molecular Dynamics, and Mechanical Properties of Poly(Ethylene Furanoate)/Poly(ε-Caprolactone) Block Copolymers

**DOI:** 10.3390/molecules29245943

**Published:** 2024-12-16

**Authors:** Johan Stanley, Panagiotis A. Klonos, Aikaterini Teknetzi, Nikolaos Rekounas, Apostolos Kyritsis, Lidija Fras Zemljič, Dimitra A. Lambropoulou, Dimitrios N. Bikiaris

**Affiliations:** 1 Laboratory of Chemistry and Technology of Polymers and Colors, Department of Chemistry, Aristotle University of Thessaloniki, GR-54124 Thessaloniki, Greece; johansta@chem.auth.gr (J.S.); pklonos@central.ntua.gr (P.A.K.); 2 Department of Physics, National Technical University of Athens, Zografou Campus, GR-15780 Athens, Greeceakyrits@central.ntua.gr (A.K.); 3 Laboratory of Advanced Materials and Devices, School of Physics, Aristotle University of Thessaloniki, GR-54124 Thessaloniki, Greece; ateknetz@physics.auth.gr; 4 Faculty of Mechanical Engineering, University of Maribor, SI-2000 Maribor, Slovenia; lidija.fras@um.si; 5 Laboratory of Environmental Pollution Control, Department of Chemistry, Aristotle University of Thessaloniki, GR-54124 Thessaloniki, Greece; dlambro@chem.auth.gr; 6 Center for Interdisciplinary Research and Innovation (CIRI-AUTH), Balkan Center, GR-57001 Thessaloniki, Greece

**Keywords:** poly(ethylene furanoate), poly(ε-caprolactone), block copolymers, thermal properties, molecular dynamics, crystallinity, mechanical properties, flexible packaging

## Abstract

This study presents the synthesis and characterization of a series of multiblock copolymers, poly(ethylene 2,5-furandicarboxylate)-poly(ε-caprolactone) (PEF-PCL), created through a combination of the two-step melt polycondensation method and ring opening polymerization, as sustainable alternatives to fossil-based plastics. The structural confirmation of these block copolymers was achieved through Attenuated Total Reflectance Fourier Transform Infrared Spectroscopy (ATR-FTIR), ensuring the successful integration of PEF and PCL segments. X-ray Photoelectron Spectroscopy (XPS) was employed for chemical bonding and quantitative analysis, providing insights into the distribution and compatibility of the copolymer components. Differential Scanning Calorimetry (DSC) analysis revealed a single glass transition temperature (*T_g_*), indicating the effective plasticizing effect of PCL on PEF, which enhances the flexibility of the copolymers. X-ray Diffraction (XRD) studies highlight the complex relationship between PCL content and crystallization in PEF-PCL block copolymers, emphasizing the need to balance crystallinity and mechanical properties for optimal material performance. Broadband Dielectric Spectroscopy (BDS) confirmed excellent distribution of PEF-PCL without phase separation, which is vital for maintaining consistent material properties. Mechanical properties were evaluated using Nanoindentation testing, demonstrating the potential of these copolymers as flexible packaging materials due to their enhanced mechanical strength and flexibility. The study concludes that PEF-PCL block copolymers are promising candidates for sustainable packaging solutions, combining environmental benefits with desirable material properties.

## 1. Introduction

Flexible packaging plays a vital role in modern packaging solutions, providing versatility and efficiency for a wide range of uses. The key attributes of flexible packaging include mechanical strength, barrier properties, and environmental sustainability. These characteristics are crucial for ensuring the packaging protects its contents effectively, remains cost-efficient, and meets environmental standards [1]. Flexible packaging for food includes a variety of materials like paper, plastic films, laminates, and aluminum foil, each offering distinct properties and uses. Plastic films, such as low-density polyethylene (LDPE) and linear low-density polyethylene (LLDPE), are widely used due to their flexibility and strength, making them ideal for various food packaging applications [2]. The growing interest in sustainable polymers for flexible packaging stems from environmental concerns and the need for eco-friendly alternatives to petroleum-based plastics, which contribute to pollution and recycling issues. Despite their promise of flexible packaging, challenges remain in optimizing their performance and reducing production costs. Continued research is essential to enhance these materials and support their wider use in the packaging industry.

Poly(ethylene furanoate) (PEF) is increasingly valued for its potential in food packaging due to its biobased source, excellent barrier performance, and strong mechanical properties [3]. Produced from renewable 2,5-furandicarboxylic acid, PEF offers a sustainable alternative to petroleum-derived plastics like poly(ethylene terephthalate) (PET) [4]. Similarly, polycaprolactone (PCL) has also been increasingly recognized for its role in sustainable food packaging due to its biodegradability and ability to blend with other polymers like PEF. Its inherent flexibility helps improve the mechanical properties of stiffer materials, providing greater impact resistance and flexibility in packaging applications. Moreover, PCL’s low melting point (about 60 °C) facilitates its processing and modification through blending or copolymerization, allowing for customization to achieve desired properties such as better barrier performance and enhanced durability [5].

Copolymers are gaining popularity in flexible packaging due to their unique properties, such as improved mechanical strength, flexibility, and barrier properties, making them ideal for various packaging applications. Styrene/butadiene block copolymers blended with polyolefins are utilized to fabricate transparent and elastic films, crucial for stretchable packaging applications. These copolymers provide an excellent balance of elasticity and transparency, making them well-suited for flexible packaging that demands both flexibility and visibility [6]. The synthesis of biobased block copolymers marks a significant advancement in sustainable packaging solutions. Bioplastics derived from poly(L-lactide) (PLLA)/poly(ethylene glycol) (PEG) block copolymers exhibit high flexibility, making them promising options for sustainable packaging. The addition of PEG as a plasticizer improves the flexibility and thermal stability of these materials, making them suitable for flexible packaging applications [7].

The incorporation of vanillic acid units into PEF to form poly(ethylene vanillate-co-ethylene furanoate) copolymers results in materials with high *T_g_* and excellent thermal stability [8]. Furan-based block copolymers synthesized from dimethyl 2,5-furandicarboxylate and dimerized fatty acid diol exhibit notable effects on thermal properties, where variations in diol chain length influence crystallization behavior and thermal transitions [9,10]. Additionally, the introduction of poly(ethylene oxide) segments in poly(butylene furanoate) copolymers promotes crystallization and phase separation, affecting key thermal properties like glass transition and melting temperatures. Additionally, the introduction of poly(ethylene oxide) segments in poly(butylene furanoate) copolymers promotes crystallization and phase separation [11].

The incorporation of comonomers such as furanoate into poly(butylene succinate) (PBS) results in a decrease in the melting temperature [12,13]. The mechanical performance of PEF copolymers is greatly enhanced by the inclusion of flexible segments such as dimerized fatty acid diol, which improves both elastic deformability and mechanical strength compared to neat PEF [9]. The balance between rigidity and flexibility, along with the environmental impact of production processes, remains an area for further investigation to fully unlock the potential of furanoate-based polyesters.

PCL plays a crucial role as a plasticizer when blended with other biodegradable polymers such as poly(lactic acid) (PLA) and poly(butylene succinate) (PBS), addressing challenges like brittleness and low thermal stability. By incorporating PCL, the flexibility and toughness of PLA are significantly improved, reducing brittleness and enhancing its thermal performance. In these blends, the PCL block acts as a plasticizer, increasing the elongation at break and making the polymer chains more flexible [14]. This plasticizing effect is also evident in PBF-block-PCL copolyesters, where PCL enhances the flexibility of the polymer, making it suitable for various packaging applications [15].

This paper focuses on the synthesis of PEF-PCL block copolymers with different mass ratios and analyzes their chemical bonding, thermal, crystallinity, molecular dynamics, and mechanical properties. The study aims to explore the potential of these copolymers as sustainable alternatives for flexible packaging, emphasizing the balance of thermal stability, mechanical strength, and flexibility required for durable and adaptable packaging solutions.

## 2. Results and Discussion

### 2.1. ATR-FTIR

**Figure 1:**
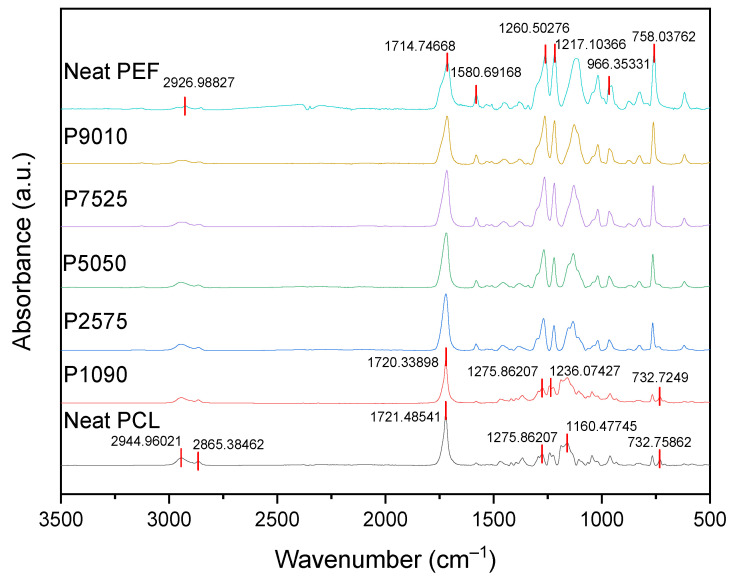
ATR-FTIR spectra of neat PEF, neat PCL, and block copolymers.

The ATR-FTIR spectra of neat PEF, neat PCL, and block copolymers are displayed in Figure 1. The neat PEF spectra provide detailed insight into the structure of PEF, highlighting the presence of ester functionalities, furan rings, and various C–H bonds. The weak bands at 3159 cm^−1^ is attributed to C–H stretching vibrations from the furan ring, while at 2926 cm^−1^ and 2873 cm^−1^ correspond to asymmetric and symmetric C–H stretching vibrations, likely from alkyl groups. The strong absorption at 1714 cm^−1^ is assigned to C=O stretching, indicating the presence of ester groups in the polymer backbone. Aromatic C=C bending vibrations are observed at 1580 cm^−1^, and C–H deformation and wagging vibrations at 1508 cm^−1^ and 1457 cm^−1^ further confirm the presence of the furan ring. Additionally, C–H rocking vibrations at 1382 cm^−1^ are likely due to methylene groups, while the peaks at 1260 cm^−1^ and 1217 cm^−1^ are attributed to C–O stretching vibrations in the ester groups. The bands at 1111 cm^−1^ and 1020 cm^−1^ arise from C–O–C ring vibrations in the furan ring. Finally, C–H out-of-plane deformation vibrations are evident at 966 cm^−1^, 827 cm^−1^, and 758 cm^−1^, with a C–H bending vibration observed at 620 cm^−1^ [16].

Similarly, neat PCL displays characteristic peaks that provide insight into its molecular structure. The peaks at 2944 cm^−1^ and 2865 cm^−1^ correspond to the asymmetric and symmetric stretching vibrations of the CH_2_ groups, respectively. The strong absorption at 1721 cm^−1^ is attributed to the ester C=O stretching, indicating the presence of ester groups in the polymer backbone. The peak at 1275 cm^−1^ is attributed to both C–O and C–C stretching vibrations, particularly in the crystalline phase of the polymer. Additionally, the peak at 1160 cm^−1^ is assigned to the ester C–O stretching vibration [17].

As depicted in Figure 1, the block copolymer spectra display all the characteristic signals of the PEF moiety. The absorption bands from the furan ring remained unchanged after copolymerization, while the ester group signals, C=O stretching (1260 cm^−1^ to 1275 cm^−1^) and C–O–C stretching (1217 cm^−1^ to 1236 cm^−1^) shifted slightly to higher wavenumbers compared to neat PEF. This shift occurs due to overlapping vibrations from the ester groups in both PEF and PCL macromolecular chains. Moreover, the appearance of a new absorption band at 732 cm^−1^ is attributed to methylene (–CH_2_–) deformation in the PCL unit; specifically, in the block copolymers. The peak shifts to higher wavenumbers and the appearance of an additional absorption band confirms the successful incorporation of ε-CL into the PEF backbone and the successful formation of copolymers [15].

### 2.2. Chemical Bonding and Quantitative Analysis (XPS)

**Figure 2:**
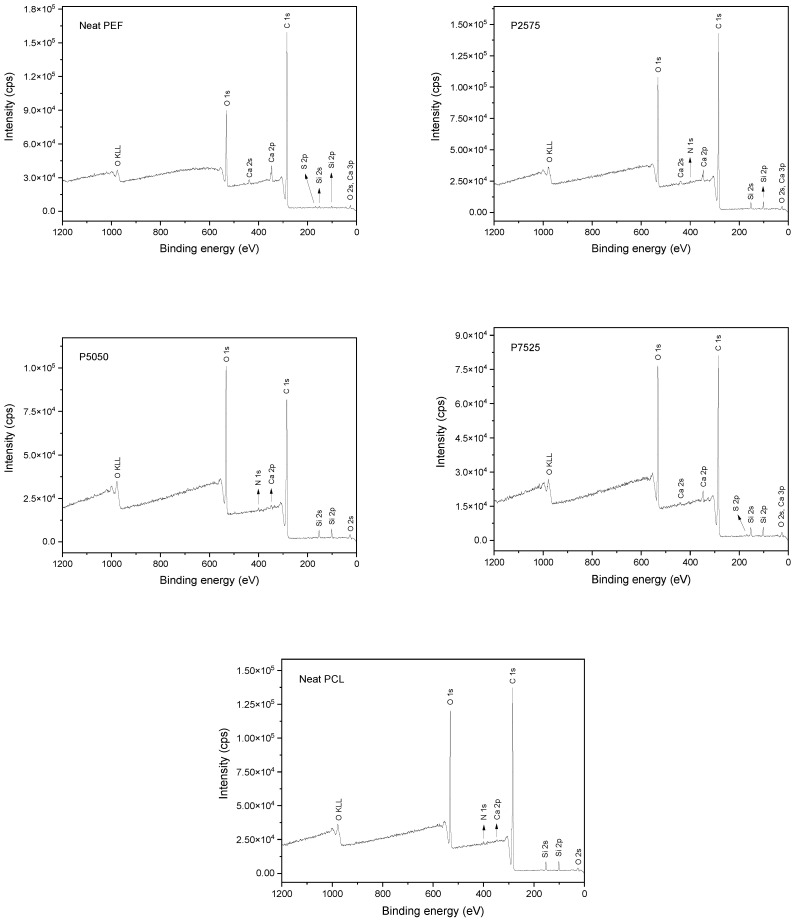
XPS survey of sample neat PEF, PCL, and their copolymers.

XPS surveys were collected to identify the elements present in the samples, as presented in Figure 2 for samples of neat PEF, P2575, P5050, P7525, and neat PCL, respectively. The primary elements detected in all samples were carbon (C) and oxygen (O), while distinct peaks of silicon (Si) and calcium (Ca) contamination (probably from the round glass bottle used for PEF synthesis) were also present. For the neat PCL, P2575, and P5050 samples, additional small peaks of nitrogen (N) contamination were observed. Finally, the surveys of P7525 and neat PEF revealed weak peaks of sulphur (S), which were also attributed to contamination.

The corresponding quantitative analyses performed on the identified elements are given for all the samples in Tables S1–S5. According to the results, the (oxygen-to-carbon) O/C ratio was calculated to be 0.22, 0.28, 0.40, 0.32, and 0.29 for neat PEF, P2575, P5050, P7525, and neat PCL, respectively. An increase in the O/C ratio was observed in copolymers due to the introduction of ε-CL into the PEF backbone but decreases as the PCL content becomes more prominent. As the proportion of PEF increases in the copolymer, the O/C ratio initially rises due to the higher oxygen content in PEF. However, as the PCL content becomes more dominant, the O/C ratio decreases, reflecting the lower oxygen content in PCL compared to PEF. The O/C ratio in copolymers is a critical parameter that indicates the presence of polar oxygen-containing functional groups such as hydroxyl, carbonyl, ester, or ether groups related to carbon atoms. Both PEF and PCL polymers contain ester groups in their polymer backbone. The ester groups in PEF are formed through the reaction between the carboxylic acid groups of 2,5-Furandicarboxylic acid (FDCA) and the hydroxyl groups of ethylene glycol, creating a repeating ester linkage along the polymer backbone. Ester groups are inherent in PCL due to their polyester nature, and their presence can be further manipulated through copolymerization with other monomers. A higher O/C ratio typically suggests an increased presence of these functional groups, which can significantly influence the physical and chemical properties of the copolymers. Copolymerizing ε-caprolactone with monomers like oxo-crown ether introduces polar groups into the polymer backbone, enhancing hydrophilicity and modifying thermal properties [18]. Literature indicates that increased oxygen content in copolymer samples can reduce crystallinity, which, in turn, affects thermal stability and mechanical properties [19]. The introduction of PCL, which contains oxygen atoms, led to changes in the polymer’s structural arrangement, contributing to reduced crystallinity. This effect is specific to the PEF-PCL copolymer system studied and may not be applicable to all oxygen-rich polymers. This reduction in crystallinity, due to oxygen incorporation leading to changes in their structural arrangement, results in the decreased rigidity of the polymers [20].

**Figure 3:**
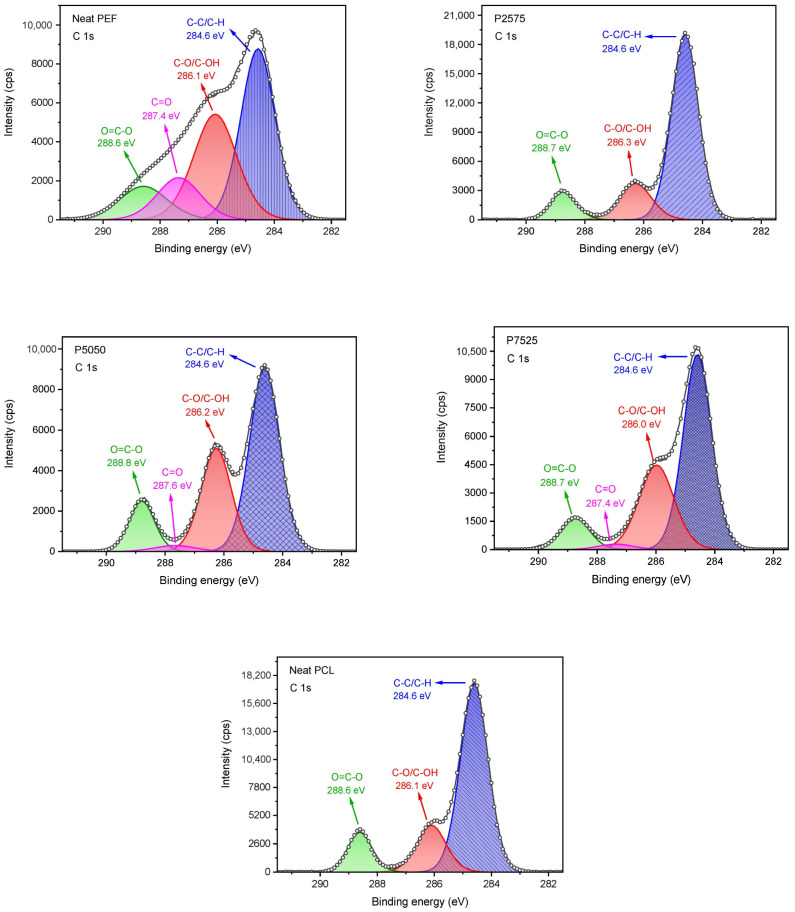
High-resolution XPS spectrum of core level C 1s for neat PEF and PCL samples and their copolymers.

The binding energies observed in high-resolution XPS spectra provide critical insights into the functional groups present in block copolymers, as seen in Figure 3 for the carbon region (C 1s). Peaks around 284.6 eV correspond to C–C and C–H bonds, representing non-polar hydrocarbon chains typically found in the aliphatic portions or backbone chains of polymers. A peak near 286.0 eV indicates C–O and C–OH bonds, which are characteristic of oxygen-bound carbons found in groups such as alcohols (–OH), ethers (R–O–R), or esters (–COO–). This often suggests the presence of oxygen-rich segments within the copolymer. At 287.4 eV, the C=O peak signifies carbonyl groups, common in ketones, aldehydes, and esters, pointing to segments with carbonyl functionality. Finally, the O=C–O bond at 288.6–289.0 eV, indicative of carboxylate or ester linkages, confirms the presence of ester functionalities, which are essential in polyester structures. In the copolymer samples, differences in the ratios of C–C/C–H, C–O/C–OH, C=O, and O=C–O bonds reveal how PEF and PCL blocks arrange themselves and influence each other.

The results in Table 1 provide insight into the distribution of carbon bonding types in various PEF-PCL block copolymer samples, analyzed through high-resolution (HR) XPS. The XPS analysis results provide a deeper understanding of the structural arrangement within the block copolymers, which is critical for evaluating how the block compositions influence material properties, particularly for flexible packaging applications. By examining the distinct carbon bonding environments, we gain insights into the distribution, compatibility, and phase behavior of PEF and PCL segments within each copolymer composition.

In the neat PEF sample, the presence of a relatively high C=O percentage (13.2%) alongside substantial C–C/C–H (42.4%) and C–O/C–OH (32.9%) bonds indicates that PEF has significant polar carbonyl (C=O) and ether/ester (C–O) functionalities. This suggests a homogeneous PEF phase with strong intra- and intermolecular interactions, which contribute to PEF’s inherent rigidity and barrier properties. The O=C–O content (11.6%) reflects ester linkages within the PEF structure, emphasizing PEF’s strong polyester characteristics.

Conversely, the neat PCL sample shows a markedly different profile, with C–C/C–H bonds dominating at 69.1%, indicating a highly aliphatic, non-polar structure. The lower C–O/C–OH (18%) and O=C–O (12.9%) content, combined with the absence of distinct C=O bonds, suggests a more flexible and less polar material, with ether and ester linkages primarily contributing to PCL’s soft, elastomeric nature. This high C–C/C–H ratio in neat PCL confirms a phase dominated by non-polar chains, which facilitates flexibility but limits thermal and mechanical performance.

P2575 sample is overwhelmingly PCL-rich, as indicated by the high C–C/C–H ratio (72.5%), with reduced C–O/C–OH (17%) and an absence of C=O, consistent with a material heavily weighted toward the flexible, non-polar PCL block. The O=C–O contribution (10.5%) indicates a limited number of ester linkages, suggesting that PEF’s influence is minor, possibly dispersed in small, isolated segments within a continuous PCL matrix. This arrangement is likely to enhance flexibility while retaining some of PEF’s thermal and mechanical characteristics due to its low but meaningful presence.

The P5050 sample has a balanced bonding profile with 54.4% C–C/C–H and 30.6% C–O/C–OH, along with small but significant contributions of C=O (2.4%) and O=C–O (12.6%). This intermediate profile suggests a well-integrated blend of PEF and PCL, where neither block forms large, distinct domains. The near-even distribution of C–C/C–H and C–O/C–OH bonding types implies miscibility or the intimate mixing of PEF and PCL segments at a molecular level, which could result in enhanced mechanical properties and moderate flexibility while maintaining some of PEF’s barrier functionality due to the preserved carbonyl groups.

The P7525 sample is dominated by PEF, as reflected by a moderate C–C/C–H content (57.6%) and higher C–O/C–OH (30.9%) bonds. The reduced C=O (2.0%) and O=C–O (9.5%) ratios imply that PEF forms the majority of the matrix, with small, dispersed PCL segments. This configuration likely retains PEF’s rigidity and barrier properties while incorporating PCL’s minor plasticizing effect. The reduced ester content suggests that PCL chains may be present in isolated or small clusters rather than forming a continuous phase, supporting the idea of a PEF-dominant matrix with minor flexibility.

The analysis of carbon bonding types helped identify the phase behavior of PEF and PCL segments, supporting the successful formation of a block copolymer structure. Lower PEF content results in a flexible, PCL-dominated matrix with isolated PEF segments that enhance barrier functionality, while higher PEF content creates a rigid matrix with dispersed PCL segments, offering limited flexibility. The balanced P5050 composition suggests a homogeneous blend, ideal for applications requiring flexibility. These insights help tailor copolymer compositions to meet specific packaging needs by effectively balancing flexibility, thermal, and mechanical properties.

### 2.3. Thermal Transitions: Glass Transition and Crystallization (DSC)

**Figure 4:**
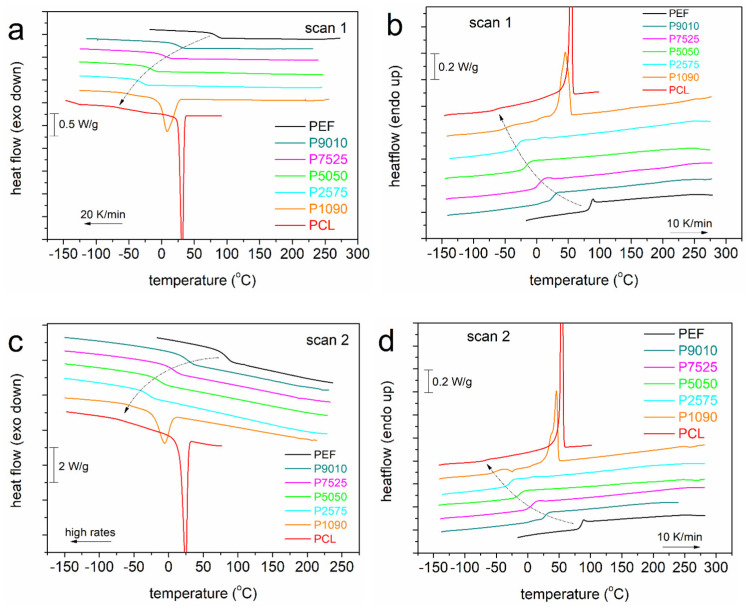
Comparative DSC traces for all samples and both scans performed. (**a**) during quenching, (**b**) subsequent heating after quenching, (**c**) cooling with 80–100 K/min and (**d**) subsequent heating after (**c**). The recorded heat flow is presented upon normalization to the mass of each sample, while the added arrows mark the recorded effects of PCL addition on the glass transition.

In Figure 4, we present the results using DSC in terms of comparative cooling and heating traces for all samples and for scans 1 and 2, respectively, involving slower (20 K/min) and faster cooling (80–100 K/min). The main thermal events recorded using DSC, i.e., crystallization, melting, and glass transition, are evaluated mainly in terms of characteristic temperatures, and the values are listed in Table 2. The fast cooling was actually performed in order to suppress or even vanish the crystallization of PCL so that the more direct effects of the structure (composition, molecular weight) can be identified. As in previous works on PCL, its crystallizability is quite strong and cannot be eliminated by conventional cooling [21,22]. Neat PCL exhibits a quite strong exothermal peak during cooling at ~25 °C originating from crystallization, which is complete, judging from the absence of ‘cold crystallization’ during the subsequent heating. The semicrystalline PCL exhibits a smooth and relatively weak glass transition step at *T_g_*~−66 °C (Table 2).

PEF does not crystallize (100% amorphous) as expected, whereas it exhibits a strong and sharp glass transition step at *T_g_* = 84 °C [23].

By a simple glance at Figure 4, it is clear that the crystallization of PCL is present only in the copolymer P1090 (at around −6 °C). The absence of PCL-related crystallization within most copolymers suggests that there is no ‘micrometric’ phase separation of PCL, as such would be necessary for the development of crystallization [24]. Regarding the crystallinity degree, *X*_c_, in PCL and P1090, this can be estimated by comparing the corresponding crystallization enthalpies (~59 J/g_PCL_ and ~44 J/g_PCL_, for PCL and P1090, respectively) with the heat of fusion for PCL, taken as 139.5 J/g according to Crescenzi et al. [25]. *X_c_* equals 42 wt.% for neat PCL and ~32 wt.% for P1090, respectively, with the uncertainty being estimated as *δX_c_* = 1 wt.%.

**Figure 5:**
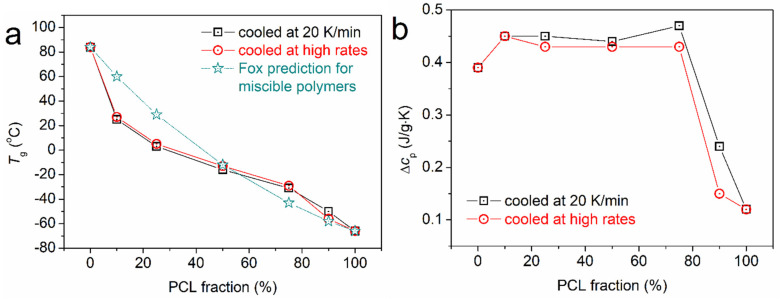
Effects on (**a**) the glass transition temperature, *T_g_*, and (**b**) the corresponding heat capacity change, Δ*c_p_*.

The most interesting effects in the copolymers refer to the glass transition. Single glass transition steps are recorded for all cases. This is a strong indication of homogeneous systems with no nanometric phase separation. Since almost all the copolymers are amorphous, we may consider that the effects on molecular mobility (*T_g_*) arise from the copolymer composition in addition to the changes in the blocks’ lengths. The *T_g_*s are shown as a function of the PCL fraction in Figure 5a. Therein, the systematic drop in *T_g_* upon the growth of the PCL blocks is observed. In Figure 5b, the strength of the glass transition, Δ*c_p_*, as a function of the PCL amount initially shows a mild increase (10% PCL), an almost stabilization (until 70%), and a sharp decrease of 90% PCL. This last change is expected, as, in P1090, there is a strong contribution of crystallization, precluding a significant amount of polymer chains (merged within the crystallites) from participating in the glass transition process.

Please note that the molecular weight values are low and expected to ‘increase’ in the copolymers with the development of the PCL blocks, inducing a tendency of *T_g_* to increase as well. The *T_g_* (PCL) trend is monotonic; however, it exhibits deviations from the predictions for well-mixed miscible polymers, e.g., the Fox prediction shown in Figure 5a [26]. This *T_g_* drop of PEF in the presence of PCL suggests a ‘plasticization-like’ effect. This is essential considering that PCL, the low *T_g_* component, could play the role of plasticizer on the ‘hard’ component (PEF) [27,28]. Also, the deviation from the Fox prediction is another indirectly supportive clue for the formation of copolymers, rather than blends.

### 2.4. Crystallinity Studies (XRD)

**Figure 6:**
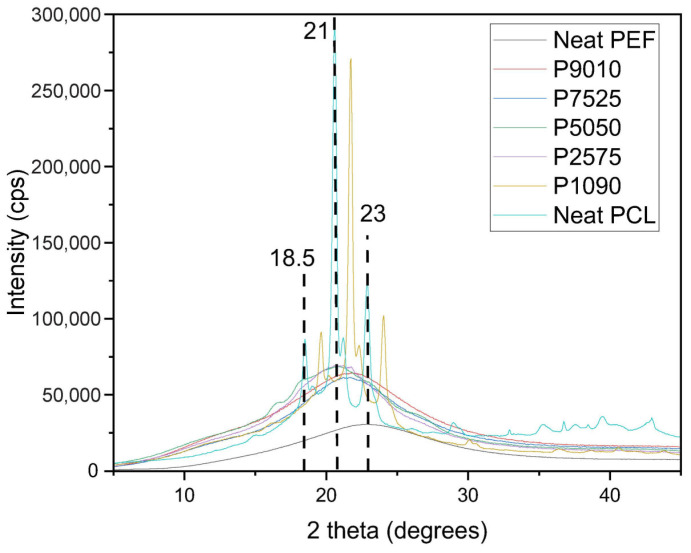
XRD patterns of amorphous neat PEF, neat PCL, and block copolymers.

The diffractograms of neat PEF, neat PCL, and all the block copolymers analyzed at room temperature are illustrated in Figure 6. The neat PEF exhibits its characteristic pattern of amorphous material, whereas the neat PCL generally displays distinct crystallization peaks, reflecting its crystalline structure. X-ray diffraction (XRD) patterns of neat PCL reveal characteristic peaks at specific 2θ angles, typically around 18.5°, 21°, and 23°, which correspond to PCL’s crystalline phases [29].

Except for PEF1090, the block copolymer with the highest PCL content, all the other block copolymers exhibited characteristic peaks of amorphous material. Based on an analysis of the complex PCL spectra [22] the degree of crystallinity, *X_c,DSC_*, was found equal to 37 ± 3% and 33 ± 3%, respectively, for PCL and P1090. Coming to the copolymers, as the PCL chain length increases, there is a corresponding rise in crystallinity and melting points, which results in a shift of the XRD peaks to higher 2θ angles [30]. The relationship between PCL content and crystallization is complex, affecting both crystallization kinetics and morphology in block copolymers. Generally, increasing the PCL content speeds up the crystallization rate of PCL blocks, as seen in several studies. For example, in poly(3-hydroxybutyrate-co-3-hydroxyvalerate)/poly(ε-caprolactone) block copolymers, higher PCL ratios resulted in faster crystallization under non-isothermal conditions [31]. Additionally, the crystallinity of PCL blocks was shown to depend on composition, with increased PCL content promoting distinct crystallization pathways [32].

However, it is important to recognize that while higher PCL content may enhance crystallization, it can also create difficulties, such as phase separation or reduced compatibility with other polymers, potentially compromising the mechanical properties [33]. These findings correlate with the DSC results.

### 2.5. Molecular Dynamics-Segmental Relaxation (BDS)

We now turn our attention to the dielectric spectroscopy results. For the sake of clarity and completeness, we should make a small introduction to the technique. In BDS, the actual molecular motions can be screened indirectly via the recording of the relaxation times and strength of corresponding dipole moments. The dipoles originate either from local or from segmental (chain-chain) motions. This method enables the study of the segmental *α* relaxation, dynamically analogous to the calorimetric glass transition [34]. The molecular relaxations are, in general, recorded as peaks in the imaginary part of dielectric permittivity, *ε’’* (dielectric loss), exhibiting maxima at some frequency, *f*. After increasing the temperature, the peaks migrate to higher frequencies due to the increasing mobility or else decrease in the corresponding relaxation times (*τ*). This is actually the recording of ‘molecular dynamics’.

**Figure 7:**
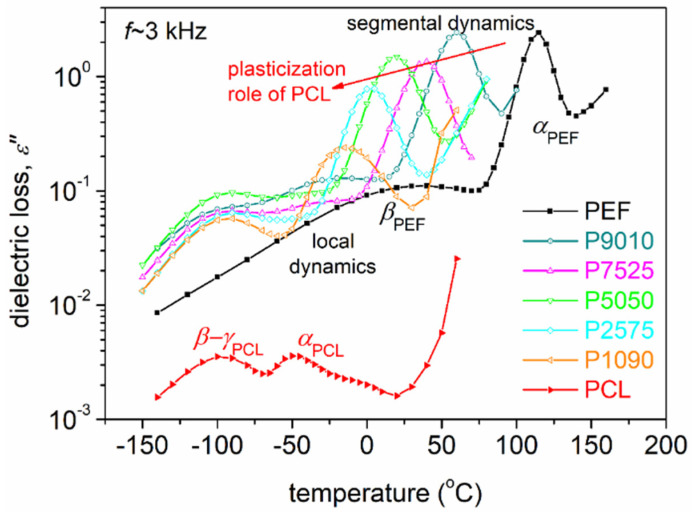
Comparative isochronal *ε*″(*T*) plots for all samples, showing the plasticization action of PCL over PEF (acceleration of the segmental relaxation, *α*).

A selection of the overall raw data is presented in Figure 7 in the form of the temperature dependence of *ε’’* at a relatively high frequency (isochronal curves). This type of presentation enables the direct comparison between the BDS and DSC data. At low temperatures, *T < T_g_*, the signal is weak, and the spectra are dominated by the contribution of dipolar local relaxations. For PEF, this is the case of the so-called β_PEF_ relaxation, originating from dipole moments related to the ester group (C=O) located close to the furan ring [35,36,37]. The ester groups are actually the most polar sites of PEF. In PCL, the local β-γ_PCL_ relaxations are recorded. The origins of the processes have not been yet strictly defined; however, there have been proposed connections to the carbonyl of PCL [38,39]. The alternations of the local relaxation between the different compositions are minor, whereas the strength of the processes is almost proportional to the fractions of PEF and PCL.

**Figure 8:**
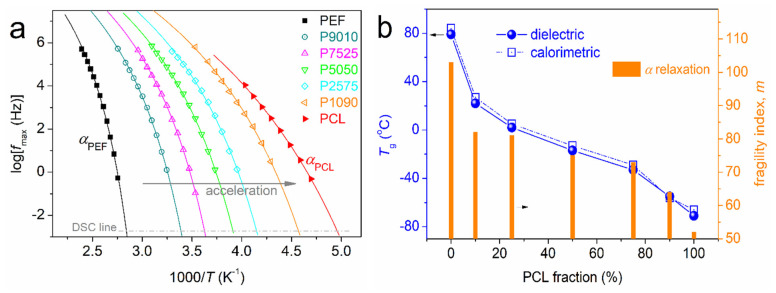
(**a**) Segmental dynamics map for all samples. (**b**) The PCL fraction effect on the dielectric/calorimetric *T*_g_ and on the fragility of *α* relaxation.

The most important results are related to the segmental dynamics. At *T ≥ T_g_*, the dielectric signal increases by one or two orders of magnitude, and the *α* relaxation dominates the response. For the homopolymers as well as the block-copolymers, we record single *α* relaxations (Figure 7). This comes in direct agreement with the single glass transitions in calorimetry and supports the concept of copolymers with no significant phase separation. Additional and more solid support could also be supplied by using small-angle X-ray spectroscopy (SAXS) combined with proper analysis [40]. By evaluating the temperature evolution of both segmental relaxations via widely used methods of analysis [41], we constructed the dynamics map shown in Figure 8a.

Compared to the copolymers, in neat PEF and PCL, the segmental relaxations are, respectively, the slowest and fastest ones. Based on the raw data and the *T_g_* changes, the presence of PCL leads to the proportional acceleration of the *α* relaxation. In all cases, the *α* relaxations demonstrate ‘curved’ time scale views in Figure 8a, which is characteristic of segmental/cooperative dynamics. The behavior is mathematically described by the Vogel–Tammann–Fulcher–Hesse (VFTH) [42] equation, which was fitted to our data (curved lines in Figure 8a). Using this fitting, we were able to estimate two crucial parameters. First, from the extrapolation of the fitted line to the equivalent frequency of DSC (log*f*~−2.8 Hz), the corresponding dielectric glass transition temperature, *T_g,diel_,* is evaluated. The data are shown in Figure 8b as a function of PCL fraction and denote the acceleration role of PCL on the segmental mobility. It is interesting that the calorimetric and dielectric data for the *T_g_*, are surprisingly similar. This is not the usual case in the literature for complex polymeric systems and suggests that there are in general no significant ‘dynamical heterogeneities’ in the PEF-PCL [43,44]. In addition, from the VFTH fitting to *α* relaxation, the so-called fragility/steepness index, *m*, is evaluated. For neat PEF, *m* equals 103, while, for neat PCL, *m* = 52. In Figure 8a, the fragility of PEF decreases systematically in the copolymers with the increasing PCL/PEF ratio. For simple polymeric systems, the fragility can be exploited as a measure of the cooperativity degree, or else can correlate with the cooperativity length [45]. In this sense, the drop in cooperativity may suggest that either the cooperativity length increases or/and the interchain distances increase. The latter is equivalent to an increase in the average ‘free volume’ when adding PCL. When this is combined with the effect of accelerated mobility (lowering of the *T_g_*), we gain quite strong evidence for the pure plasticizing role of PCL over PEF [46].

Regarding applications, both the ‘softening’ of the polymeric matrix in addition to the increasing of free volume can be of use in manipulating permeability and elasticity. Such aspects fit within various applications, for example, in packaging, gas sensor membranes, etc.

**Figure 9:**
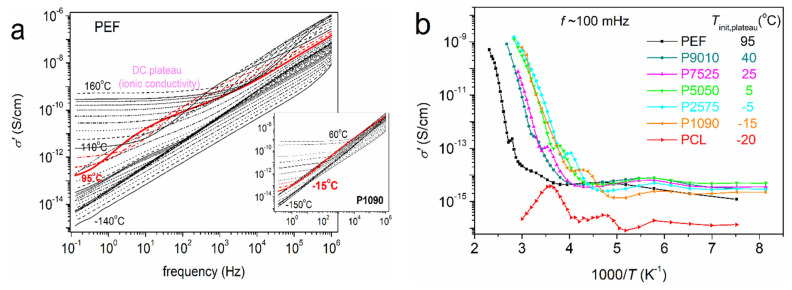
(**a**) Isothermal curves of the AC conductivity, *σ*’(*f, T*), for neat PEF and P1090, showing that the formation of DC plateau due to ionic conductivity occurs uniquely at *T* > *T*_g_. (**b**) Comparative curves of *σ*’ at the lowest frequency of recording against the reciprocal temperature (Arrhenius plots of conductivity) for all samples.

A final comment on BDS refers to the conductivity, σ’, which can be followed in Figure 9, in terms of raw data [σ’(*f*,*Τ*)] at the selected examples of PEF and P1090 (Figure 9a). To provide an overall view of all compositions, we also created the reciprocal temperature evolution of σ’ at the lowest frequency of measurement [log (σ’(*Τ*) at 0.1 Hz, Figure 9b]. At *T > T_g_*, the polymers usually exhibit a weak DC plateau (10^−11^–10^−5^ S/cm) in the σ’(*f*,*Τ*) spectrum. Such plateau denotes the transport of free charges (ions) throughout the polymer matrix, which occurs in the ‘elastic/liquid’ phase. The existence of such a plateau also denotes the continuity of this phase in the overvolume. Such is the case of neat PEF and PCL. Here, in the copolyesters, there is an absence of a DC plateau arising from PCL. Thus, a continuous PCL phase throughout the copolymer matrix seems to not exist. This provides another indication, supporting the previous findings on the homogeneous distribution of PEF-PCL entities, and, thus, it is not worth noting phase separation.

### 2.6. Mechanical Properties

**Figure 10:**
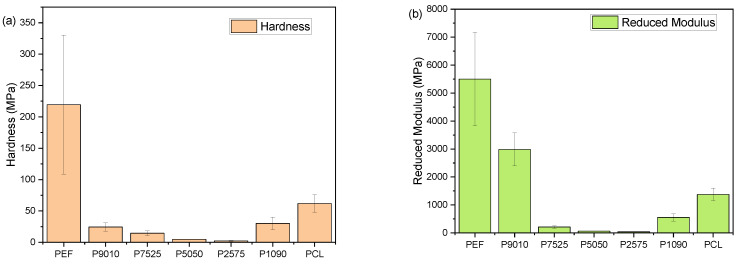
(**a**) Hardness and (**b**) reduced modulus values of block copolymers with standard deviation.

The nanoindentation test results, shown in Figure 10, display a significant standard deviation, which is likely attributed to the small surface area of the individual indents. The neat PEF exhibited a higher hardness value of (219.4 ± 110.8 MPa) and a reduced modulus value of (5500 ± 1673.5 MPa). The limited chain mobility in PEF, resulting from its molecular structure, plays a key role in enhancing its mechanical strength [47]. The neat PCL displays a hardness value of (61.9 ± 13.9 MPa) and a reduced modulus value of (1374 ± 225.7 MPa).

The addition of ε-CL into PEF polyesters decreases the hardness and elastic modulus values of all the block copolymer samples. Incorporating ε-CL into block copolymers typically leads to reduced hardness and modulus, as demonstrated in several studies [48,49,50]. This phenomenon is mainly attributed to the plasticizing effect of PCL, which increases flexibility and decreases rigidity within polymer matrices. The influence of PCL on mechanical properties is affected by factors including its molecular weight and concentration in the blend. Rahmatabadi et al. showed that incorporating PCL into PVC leads to rubbery behavior at room temperature, characterized by increased elongation and flexibility, which further contributes to the observed reduction in hardness and modulus values [51]. Jing et al. explained that the addition of PCL into PLLA/PCL copolymers enhances elongation at break and toughness, even though it may result in a decrease in tensile strength [52].

**Figure 11:**
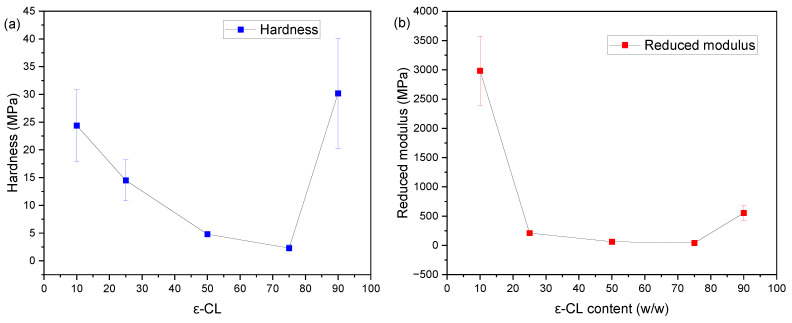
The decrease in (**a**) hardness and (**b**) reduced modulus values with respect to ε-CL content.

The trend of decrease in hardness and reduced modulus values with respect to the ε-CL has been displayed in Figure 11. However, a slight improvement in hardness and reduced modulus values have been observed in the PEF1090 sample. This may be attributed to an increase in the crystallinity of the sample as observed in DSC and XRD measurements. Typically, an increase in crystallinity results in higher hardness and modulus values in copolymers. This correlation is mainly attributed to the improved structural integrity and resistance to the deformation that crystallinity provides [53].

The balance between reduced mechanical strength and enhanced flexibility can be beneficial in certain packaging applications, such as sustainable flexible packaging, as they directly impact the durability and adaptability of packaging materials. Guidotti et al. fabricated furan-based multiblock copolymer containing PEG-like sequences as biobased and compostable films for sustainable flexible packaging applications. These films exhibited improved elongation at break and lower elastic modulus, contributing to their flexibility [54].

## 3. Materials and Methods

### 3.1. Reagents and Solvents

2,5-Furandicarboxylic acid (BioFDCA X000230-2003) was purchased from Corbion, Gorinchem, the Netherlands. Ethylene glycol (anhydrous, 99.8%) and antimony trioxide (Sb_2_O_3_) were purchased from Sigma–Aldrich Co., London, UK. ε-Caprolactone (ε-CL, purity 99%) and stannous octoate (Sn(Oct)_2_) used as catalyst were purchased from Sigma–Aldrich, Saint Louis, MO, USA. All organic solvents were used without further purification.

### 3.2. Synthesis of Neat PEF and PEF-PCL Block Copolymers

**Scheme 1:**
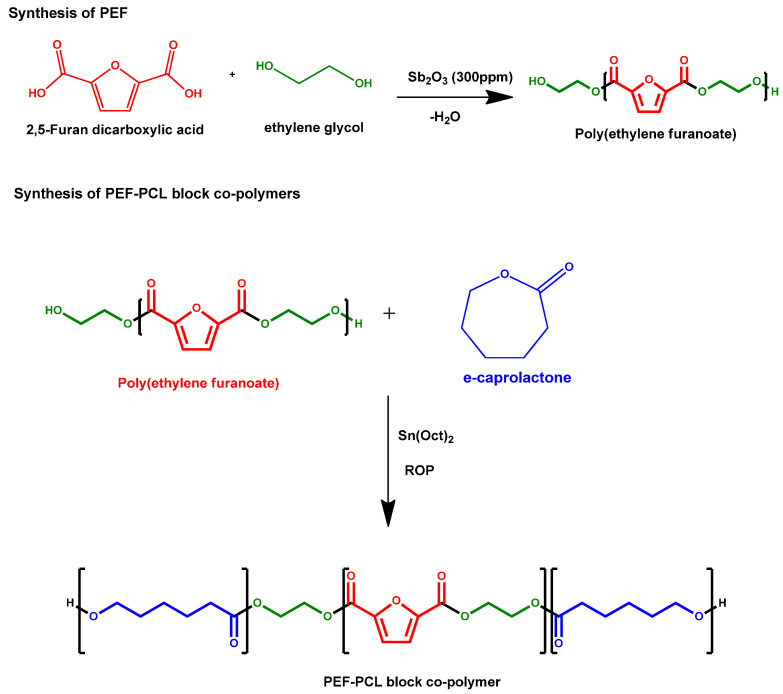
Synthesis route to PEF and PEF-PCL block copolymers.

Neat PEF polyester was synthesized using a two-stage melt polycondensation method using FDCA and ethylene glycol (EG) utilized at a 1:2.1 molar ratio as per our previous study [16]. PEF/PCL block copolymers with various mass ratios (10/90, 25/75, 50/50, 75/25, and 90/10 *w*/*w*) were synthesized by ring-opening polymerization (ROP), which typically does not require solvents as it is conducted in a melt state. The synthesis route of PEF and PEF-PCL block copolymers has been illustrated in Scheme 1. Appropriate ratios of PEF and ε-CL monomers were added, as displayed in Table 3. The ring-opening polymerization of ε-CL took place at 200 °C under nitrogen flow with a stirring rate of 300 rpm. In general, ε-CL is known to polymerize well at lower temperatures, often around (100–150) °C. However, when copolymerizing, higher temperatures were necessary to facilitate the reaction and help in achieving a balance between polymer chain mobility and crystallization, leading to desirable material properties [55]. Based on the literature, The kinetic model for ROP suggests that higher temperatures, typically between 150 °C and 220 °C, can lead to faster monomer conversion, which is essential for achieving high molecular weights and efficient polymerization in the short period of time [56,57,58]. Sn(Oct)_2_ was introduced as a catalyst (0.5 wt.% of the total materials) prior to the polycondensation step, and the reaction was allowed to proceed for 2.5 h. The resulting block copolymers were purified to remove the unreacted monomers by dissolving them into trifluoroacetic acid and then precipitated in cold methanol prior to characterization.

### 3.3. Characterization

#### 3.3.1. Nuclear Magnetic Resonance (NMR)

The copolymers were dissolved in a mixture of deuterated chloroform (CDCl_3_) and deuterated trifluoroacetic acid (TFA-d_1_). NMR spectra were obtained using an Agilent 500 spectrometer (Agilent Technologies, Santa Clara, CA, USA), with calibration based on the residual solvent peaks of CDCl_3_.

#### 3.3.2. Attenuated Total Reflectance Fourier Transform Infrared Spectroscopy (ATR-FTIR)

The ATR spectra of all the samples were acquired using an IRTracer-100 (Shimadzu, Japan) with a QATR™ 10 Single-Reflection ATR Accessory and a Diamond Crystal. The spectra were obtained from 400 to 4000 cm^−1^ at a resolution of 2 cm^−1^ (total of 16 co-added scans), with the baseline corrected and transformed to absorbance mode.

#### 3.3.3. X-Ray Photoelectron Spectroscopy (XPS)

X-Ray Photoelectron Spectroscopy (XPS) measurements were performed using a Kratos Analytical AXIS Ultra^DLD^ system (Kratos Analytical, Manchester, UK), equipped with an aluminum monochromatic X-ray source (λ_Ka_ = 1486.6 eV). Survey spectra (0–1200 eV) were collected with an analyzer pass energy of 160 eV. High-resolution (HR) spectra were acquired with a pass energy of 20 eV over three-sweep scans to ensure enhanced accuracy. The charging effect was calibrated using the C 1s peak at 284.6 ± 0.2 eV from C–C bonds. The background of the HR spectra was subtracted using Shirley (non-linear) baselines, while the experimental curves were optimally fitted with a combination of Gaussian (70%) and Lorentzian (30%) distributions. Relative Sensitivity Factors (RSFs) for each element were obtained from the Vision 2.2.10 software database.

#### 3.3.4. Differential Scanning Calorimetry (DSC)

Thermal transitions were analyzed using a TA Q200 DSC instrument (TA Instruments, New Castle, DE, USA), calibrated with sapphires for heat capacity and indium for temperature and enthalpy, on samples weighing approximately 7–8 mg sealed in aluminum TZero TA pans, within a temperature range from −150 °C to 260 °C under a high-purity helium atmosphere (99.9995%). The polymers were first heated to 260 °C to remove any thermal history and ensure good thermal contact with the pan, followed by two cooling-heating cycles: a conventional cooling (scan 1, 20 K/min) and a faster cooling (scan 2, 80–100 K/min) in the expected crystallization region, to produce amorphous and semi-crystalline polymers, respectively. Subsequential heating scans were then recorded at a fixed rate of 10 K/min.

A PerkinElmer Pyris DSC-6 differential scanning calorimeter, (PerkinElmer Inc., Waltham, MA, USA), calibrated with pure indium and zinc standards, was used to measure annealed samples of 5 ± 0.1 mg in sealed aluminum pans under an N_2_ atmosphere at a flow rate of 20 mL/min.

#### 3.3.5. X-Ray Diffraction (XRD)

The XRD spectra of all the samples were obtained using MiniFlex II XRD equipment (Rigaku Co., Tokyo, Japan), using Cu Ka radiation (0.154 nm) spanning a 2θ range from 5° to 50°, with a scanning rate of 1°/min.

#### 3.3.6. Broadband Dielectric Spectroscopy (BDS)

The study of molecular dynamics, with a focus on segmental mobility, was conducted using Broadband Dielectric Spectroscopy (BDS) (Novocontrol Technologies, Montabaur, Germany) [34], under a nitrogen atmosphere, employing a Novocontrol Alpha Frequency Response Analyzer (Novocontrol Technologies, Montabaur, Germany), and a Quatro Liquid Nitrogen Cryosystem (Novocontrol Technologies Montabaur, Germany). Measurements were taken on initially amorphous samples, prepared by melting and fast cooling, arranged in a Sandwich-like Capacitor (Novocontrol Technologies Montabaur, Germany), with a 20 mm diameter and ~1.2 mm electrode distance. The complex dielectric permittivity (ε* = ε’ − i·ε″) was recorded isothermally over a frequency range from 10^−1^ to 10^6^ Hz and a temperature range from −150 °C to 160 °C, with heating steps of 5 or 10 °C depending on the process being monitored. The results are evaluated and discussed in terms of time scale (dynamics maps), dielectric glass transition temperature, and fragility index of the segmental [41].

#### 3.3.7. Nanoindentation Testing

Nanoindentation tests were carried out utilizing a Nano Test Vantage (Micro Materials Limited, Wrexham, UK). The data depth calibration has been adjusted to 1985.5 nm/Bit, whereas the load calibration has been adjusted to 74.8 µN/Bit. A Berkovich Indenter, CSM Instruments (now part of Anton Paar) (Peseux, Switzerland), was used to measure the following indentation properties: hardness, elastic modulus, plastic work, and elastic work. The loading time was 10 s up to a maximum load of 20 mN, 3 s at maximum load, and 1 s to unload. A total of 16 measurements were conducted on the basic polymers, and 25 on the copolymers with a spacing of 50 µm.

## 4. Conclusions

In conclusion, the synthesis of poly(ethylene 2,5-furandicarboxylate)-poly(ε-caprolactone) (PEF-PCL) multiblock copolymers through a two-step melt polycondensation method has successfully integrated ε-CL into the PEF backbone, as confirmed by ATR-FTIR analysis. XPS data indicated an increase in the O/C ratio, reflecting a greater presence of functional groups that reduce crystallinity and influence thermal and mechanical properties. Notably, the P5050 composition demonstrates a homogeneous blend, which is advantageous for applications that require a balance of flexibility and durability. Much strong evidence is collected on the formation of quite homogeneous systems and, indeed, of successful copolymeric synthesis. PCL acts clearly as a plasticizer on PEF, which can be exploitable regarding applications, processing, and, moreover, composability. Nanoindentation measurements showed a reduction in hardness and modulus with increased ε-CL content, with a slight improvement in the highly crystalline PEF1090. Overall, these findings suggest that the PEF-PCL copolymers provide an effective balance of thermal stability, mechanical strength, and flexibility, making them promising candidates for sustainable flexible packaging applications where durability and adaptability are essential.

## Figures and Tables

**Table 1:** Chemical bonding’s of samples on the surface analyzed using XPS.

Sample	C–C/C–H (%)	C–O/C–OH (%)	C=O (%)	O=C–O (%)
Neat PEF	42.4	32.9	13.2	11.6
P2575	72.5	17	-	10.5
P5050	54.4	30.6	2.4	12.6
P7525	57.6	30.9	2.0	9.5
Neat PCL	69.1	18	-	12.9

**Table 2:** Samples and main calorimetric values: glass transition temperature, *T_g_*, heat capacity change during glass transition, Δ*c_p_*, crystallization, and melting temperatures, *T_c_* and *T_m_*, respectively.

Sample (mol/mol)	*T_g_* (^°^C)	Δ*c_p_* (J/g∙K)	*T_c_* (^°^C)	*T_m_* (^°^C)
PEF	84	0.39	-	-
P9010	25	0.45	-	-
P7525	3	0.45	-	-
P5050	−16	0.44	-	-
P2575	−31	0.47	-	-
P1090	−50	0.24	−6	46
PCL	−66	0.12	25	53

**Table 3:** The appropriate ratios of PEF and ε-CL are used for synthesizing block copolymers.

Sample	PEF/ ε-CL Ratio ^a^	Composition ^b^ (%)	
		PEF segments	PCL segments
PEF	100/0 (neat PEF)	-	-
P9010	90/10	87	13
P7525	75/25	74	26
P5050	50/50	52	48
P2575	25/75	24	76
P1090	10/90	8	92
PCL	0/100 (neat PCL)	-	-

^a^ Based on the feed ratio of the monomers. ^b^ As determined from the ^1^H NMR spectrum.

## Data Availability

Data are contained within the article or Supplementary Materials. The original contributions presented in this study are included in the article/Supplementary Materials. Further inquiries can be directed to the corresponding author.

## Supplementary Materials

The following supporting information can be downloaded at: https://www.mdpi.com/article/10.3390/molecules29245943/s1, Table S1: Quantification report of sample PCL neat; Table S2: Quantification report of sample PEF2575; Table S3: Quantification report of sample P5050; Table S4: Quantification report of sample P7525; Table S5: Quantification report of sample PEF neat.
