# Supplementary material for: Thermal, Molecular Dynamics, and Mechanical Properties of Poly(Ethylene Furanoate)/Poly(ε-Caprolactone) Block Copolymers
Source: Molecules. 2024 Dec 16;29(24):5943. doi: 10.3390/molecules29245943 (PMC11678855; doi:10.3390/molecules29245943)
Supplement: Supplementary file 1 [file molecules-29-05943-s001.zip › molecules-3330246-supplementary.pdf]

molecules

MDPI

Supplementary Materials

Thermal, Molecular Dynamics, and Mechanical Properties of Poly(ethylene furanoate)/poly(ε-caprolactone) block co-polymers

Johan Stanley[1], Panagiotis A. Klonos[1,2], Aikaterini Teknetzi[3], Nikolaos Rekounas[2], Apostolos Kyritsis[2], Lidija Fras Zemljič [4], Dimitra A. Lambropoulou [5,6] and Dimitrios N, Bikiaris[1,*]

[1] Laboratory of Chemistry and Technology of Polymers and Colors, Department of Chemistry, Aristotle University of Thessaloniki, GR-54124 Thessaloniki, Greece, johansta@chem.auth.gr (J.S.), pklonos@central.ntua.gr (P.A.K.)
[2] Department of Physics, National Technical University of Athens, Zografou Campus, 15780, Athens, Greece, nikolasrekounas@gmail.com (N.R.), akyrits@central.ntua.gr (A.K.)
[3] Laboratory of Advanced Materials and Devices, School of Physics, Aristotle University of Thessaloniki, GR-541 24 Thessaloniki, Greece, ateknetz@physics.auth.gr (A.T.)
[4] Faculty of Mechanical Engineering, University of Maribor, SI-2000 Maribor, Slovenia. lidija.fras@um.si (L.F.Z.)
[5] Laboratory of Environmental Pollution Control, Department of Chemistry, Aristotle University of Thessaloniki, GR–541 24 Thessaloniki, Greece; dlambro@chem.auth.gr (D.A.L)
[6] Center for Interdisciplinary Research and Innovation (CIRI-AUTH), Balkan Center, GR-570 01 Thessaloniki, Greece
* Correspondence: dbic@chem.auth.gr. (D.N.B)

Abstract: This study presents the synthesis and characterization of a series of multiblock copolymers, poly(ethylene 2,5-furandicarboxylate)-poly(ε-caprolactone) (PEF-PCL), created through a two-step melt polycondensation method aimed at flexible packaging applications. The successful formation and chemical bonding's of the copolymers revealing highly homogeneous systems were analyzed using spectroscopy techniques. DSC analysis indicated a single glass transition temperature and a notable reduction in PCL crystallinity, highlighting the plasticizing effect of PCL on PEF, which systematically reduced the glass transition temperature (T_g). Dielectric spectroscopy corroborated these findings, mapping molecular dynamics and confirming excellent PEF-PCL distribution without phase separation. Alongside variations in molecular weight, thermal and dielectric properties strongly affirm the successful synthesis of PEF-PCL copolymers. The balanced thermal stability and mechanical performance of these materials suggest that they hold promise as sustainable alternatives to fossil-based plastics, especially for flexible packaging applications.

Keywords: Poly(ethylene furanoate); Poly(ε-caprolactone); Block co-polymers; Thermal properties; Molecular Dynamics; Crystallinity; Mechanical properties; Flexible packaging.

Citation: To be added by editorial staff during production.

Academic Editor: Firstname Lastname

Received: date
Revised: date
Accepted: date
Published: date

Copyright: © 2024 by the authors. Submitted for possible open access publication under the terms and conditions of the Creative Commons Attribution (CC BY) license (https://creativecommons.org/licenses/by/4.0/).

S.1. Results and Discussion

Table S1. Quantification report of sample PCL neat.

Peak	Type	Position BE (eV)	FWHM (eV)	Raw Area (Cps eV)	RSF	Atomic Mass	Atomic Conc %	Mass Conc %
**C 1s**	Reg	284.6	1.2	31106.7	0.3	12	74.7	67
**O 1s**	Reg	531.7	2.3	27424	0.8	16	21.8	26
**Si 2p**	Reg	101.6	1.3	1254	0.3	28	2.6	5.6
**Ca 2p**	Reg	347.2	1.3	597.3	1.8	40	0.2	0.6
**N 1s**	Reg	400	1.2	451.1	0.5	14	0.6	0.6

Table S2. Quantification report of sample PEF2575.

Peak	Type	Position BE (eV)	FWHM (eV)	Raw Area (Cps eV)	RSF	Atomic Mass	Atomic Conc %	Mass Conc %
**C 1s**	Reg	284.6	1.1	30536.8	0.2	12	75	66.7
**O 1s**	Reg	531.8	2.3	26355.4	0.7	16	21.4	25.4
**Si 2p**	Reg	101.6	1.2	958.3	0.3	28	2	4.3
**Ca 2p**	Reg	346.9	1.4	2956.5	1.8	40	1.1	3.2
**N 1s**	Reg	399.1	0.8	265	0.5	14	0.4	0.4

Table S3. Quantification report of sample P5050.

Peak	Type	Position BE (eV)	FWHM (eV)	Raw Area (Cps eV)	RSF	Atomic Mass	Atomic Conc %	Mass Conc %
**C 1s**	Reg	284.6	1.3	21839.6	0.3	12	69	60.8
**O 1s**	Reg	531.7	3	26621.5	0.8	16	27.8	32.7
**Si 2p**	Reg	101.8	1.3	875.7	0.3	28	2.4	5
**Ca 2p**	Reg	347.2	1.3	718.8	1.8	40	0.3	1
**N 1s**	Reg	400	0.8	228	0.5	14	0.4	0.4

Table S4. Quantification report of sample P7525.

Peak	Type	Position BE (eV)	FWHM (eV)	Raw Area (Cps eV)	RSF	Atomic Mass	Atomic Conc %	Mass Conc %
**C 1s**	Reg	284.5	1.4	21991.5	0.3	12	73	64
**O 1s**	Reg	531.8	2.5	21131.2	0.8	16	23.2	27.1
**Si 2p**	Reg	101.8	1.5	858.2	0.3	28	2.5	5.2
**Ca 2p**	Reg	347	1.8	2169.7	1.8	40	1	3.2
**N 1s**	Reg	168	1.3	146.3	0.7	32	0.2	0.5

Table S5. Quantification report of sample PEF neat.

Peak	Type	Position BE (eV)	FWHM (eV)	Raw Area (Cps eV)	RSF	Atomic Mass	Atomic Conc %	Mass Conc %
**C 1s**	Reg	284.7	3.1	31719.7	0.3	12	80	72
**O 1s**	Reg	531.5	2	21046.1	0.8	16	17.5	21
**Si 2p**	Reg	102.1	2	361.1	0.3	28	0.8	1.7
**Ca 2p**	Reg	346.6	2.1	4047.9	1.8	40	1.5	4.6
**N 1s**	Reg	168.2	1.4	253.2	0.7	32	0.3	0.7

Author Contributions: "Conceptualization, methodology, formal analysis, investigation, J.S., P.A.K., A.T., N.R., A.K., L.F.Z., D.A.L., and D.N.B.; writing—original draft preparation, J.S., P.A.K., A.T., A.K., and D.N.B.; writing—review and editing, J.S., P.A.K., A.T., N.R., A.K., L.F.Z., and D.N.B.; supervision, D.N.B., L.F.Z., and D.A.L.; project administration, D.N.B., L.F.Z., and D.A.L.; funding acquisition, D.N.B., L.F.Z., and D.A.L. All authors have read and agreed to the published version of the manuscript.

Funding: The authors acknowledge financial for this work from the EU Horizon 2020 research and innovation program under the MSCA FoodTraNet project (grant agreement no. 956265).

Institutional Review Board Statement: Not Applicable.

Informed Consent Statement: Not Applicable.

Data Availability Statement: No new data were created or analyzed in this study. Data sharing is not applicable to this article.

Acknowledgments: The authors acknowledge the use of research equipment "Hardness, micro-hardness and Indentation Tester" procured within the operation "Upgrading national research infrastructures – RIUM", which was co-financed by the Republic of Slovenia and the European Union from the European Regional Development Fund, and the Laboratory for materials, UM FS, and Franc Zupanič for support by measurements.

Conflicts of Interest: The authors declare no conflicts of interest.

Disclaimer/Publisher's Note: The statements, opinions and data contained in all publications are solely those of the individual author(s) and contributor(s) and not of MDPI and/or the editor(s). MDPI and/or the editor(s) disclaim responsibility for any injury to people or property resulting from any ideas, methods, instructions or products referred to in the content.
